# *Wushenziye* Formula Inhibits Pancreatic *β* Cell Apoptosis in Type 2 Diabetes Mellitus via MEK-ERK-Caspase-3 Signaling Pathway

**DOI:** 10.1155/2018/4084259

**Published:** 2018-09-25

**Authors:** Chunyu Tian, Hong Chang, Xiaojin La, Ji-an Li, Leilei Ma

**Affiliations:** ^1^North China University of Science and Technology, Tangshan 063210, China; ^2^Pharmacology Analysis Key Laboratory for Prevention and Treatment of Diabetes of Traditional Chinese Medicine in Hebei Province, Tangshan 063210, China

## Abstract

**Background:**

* Wushenziye* formula (WSZYF), composed of* Radix Polygoni Multiflori Preparata*,* Mori fructus*,* Mori folium*, and* Cassiae semen*, is effective in the treatment of type 2 diabetes mellitus (T2DM).

**Aim:**

In this study, we aimed to explore the effects and the underlying mechanisms of WSZYF on inhibiting pancreatic *β* cell apoptosis and improving insulin resistance (IR) in T2DM.

**Methods:**

A T2DM model was induced by Goto-Kakizaki diabetes prone rats. Cell apoptosis model was induced in MIN6 cells.

**Results:**

* In vivo*, WSZYF decreased fasting blood glucose (FBG), insulin concentration, insulin resistance index, triglyceride (TG), total cholesterol (TC), and free fatty acids (FFA) in T2DM rats. Meanwhile, WSZYF ameliorated impairments in the morphology and structure of pancreatic tissues.* In vitro*, WSZYF enhanced cell viability and promoted insulin secretion in the apoptosis model of MIN6 cells. Furthermore, WSZYF modulated the expressions of apoptosis-related molecules by increasing the expressions of MEK1/2, p-MEK1/2, ERK1/2, and p-ERK1/2 and decreasing the cleaved-caspase-3 expression.

**Conclusion:**

These findings indicate that WSZYF may become a new drug candidate in the treatment of T2DM and its antidiabetic mechanism is probably inhibiting pancreatic *β* cell apoptosis by modulating the MEK-ERK-Caspase-3 signaling pathway.

## 1. Introduction

With the change of dietary habits and improvement of living standards, the incidence of diabetes mellitus rising and the number of global diabetes in 2030 are expected to be 3 times more than those in 2000 [1]. Type 2 diabetes mellitus (T2DM) is an endocrine and metabolic disease characterized by hyperglycemia. Dysfunction of pancreatic *β* cell plays an important role in the pathogenesis of T2DM [2]. Insulin resistance (IR) occurs in most T2DM patients and body sensitivity to insulin decreases. Meanwhile, the insulin secretion will compensate for IR. However, compared with hyperglycemia, the higher level of insulin is still deficient. The function of pancreatic *β* cell is gradually damaged by the long-term high level of insulin secretion [3]. Study showed that impaired function and increased apoptosis of pancreatic *β* cell were present at the early stage of T2DM and the damage was exacerbated after the onset of the disease [4]. As the course of the disease progresses, the function of islet is decompensated, manifested by the reduction of pancreatic *β* cell replication, volume, regeneration, and apoptosis [5]. Therefore, the dysfunction and apoptosis of pancreatic *β* cell are the main causes for the decrease of insulin secretion. Pancreatic *β* cell protection, apoptosis reduction, and insulin secretion improvement are the key points in the treatment of T2DM.

Methyl ethyl ketone (MEK)/extracellular regulated protein kinases (ERK) is the main member of mitogen activated protein kinase (MAPK) families, while the role of the same signal pathway varies in different cells and situations [6]. It has been reported that MEK/ERK signaling pathway plays an important role in controlling cell proliferation, differentiation, and apoptosis. Previous experiments have demonstrated that the expression of MEK/ERK could protect cells, promote proliferation, and prevent cell apoptosis [7, 8]. Reduction of the ERK pathway in islet and MIN6 cells was associated with apoptosis [9, 10].

In Traditional Chinese medicine, a great deal of valuable experience in the prevention of T2DM has been accumulated. Chinese herbal compound is characterized by multiple components and multiple targets, thus regulating the metabolism of sugar and fat in T2DM as a whole. It can not only increase the number of insulin receptors, but also regulate the signal transduction and prevent and control the occurrence and development of diabetic complications [11].* Wushenziye* formula (WSZYF), composed of* Radix Polygoni Multiflori Preparata, Mori fructus, Mori folium,* and* Cassiae semen*, is effective in the treatment of T2DM. Our previous study indicated that the formula prevented T2DM via improving IR in skeletal muscle and reducing blood sugar [12]. However, the underlying mechanism and effective components are still unclear. In this study, a T2DM rat model and an apoptosis model of MIN6 cells were established to investigate whether WSZYF could treat T2DM by inhibiting pancreatic *β* cell apoptosis and its role in regulating MEK-ERK-Caspase-3 signaling pathway.

## 2. Materials and Methods

### 2.1. Materials

Insulin assay kit was obtained from Millipore (USA). Dulbecco's modified Eagle's medium (DMEM), phenol red-free DMEM, and fetal bovine serum (FBS) were from Gibco (USA). Penicillin, streptomycin, and 0.25% trypsin were purchased from Invitrogen (USA). The Annexin V-FITC&PI kit was obtained from Merck Millipore (USA). The 3-(4,5)-dimethylthiahiazo(-z-y1)-3,5-diphenytetrazoliumromide (MTT) was obtained from Sigma (USA). Metformin was purchased from Squibb Pharmaceutical (Shanghai, China). Blood sugar meter and blood sugar test paper were purchased from Sannuo Biosensors (Changsha, China). Assay kits of triglyceride, total cholesterol, and free fatty acids were from Nanjing Jiancheng Bioengineering Institute (Nanjing, China). Palmitic acid was from Sigma (USA).The primary antibodies against ERK1/2, p- ERK1/2, MEK1/2, p-MEK1/2, cleaved-caspase-3, and GAPDH were from Abcam (USA). The secondary antibodies were purchased from Beyotime Biotechnology (China). The herbs in WSZYF were purchased from Tongrentang Pharmacy (Tangshan, China).* Wushenziye* formula (freeze-dried powder) dissolved in DMEM was deposited in -20°C.

### 2.2. The Preparation of WSZYF and the Chemical Analysis of WSZYF by HPLC

Freeze-dried powder of WSZYF was prepared by Traditional Chinese Medical College, North China University of Science and Technology. Freeze-dried powder of WSZYF was weighed accurately (0.04 g) and placed into a 1.5 ml centrifuge tube containing 1.0 ml 50% aqueous methanol for 5 minutes in a vortex. Following centrifugation at 12000 rpm for 10 minutes in a centrifuge (Eppendorf, Melbourne, Australia), supernatant (200 *μ*l) was 5-fold diluted with 50% aqueous methanol and centrifuged at 12000 rpm for 10 minutes.

The HPLC/MS analysis was carried out on a Waters Quattro Premier XE, ESI, and Mass Lynx V4.1 workstation. The chromatographic separation was performed on a Waters BEH C18 column (2.1×100 mm, 1.7 *μ*m). HPLC determination conditions: mobile phase, 0.1% formic acid water (A) - acetonitrile (B), gradient elution (0~2 min: 1% ~5% B; 2~5 min: 6% ~15%B; 5~7 min: 16% ~25 % B; 7~13.5 min: 26 % B; 13.5~14.5 min: 30% ~ 60% B; 14.5~16 min: 70% B). Column temperature: 50°C, injection volume: 5 *μ*L; flow rate: 0.3mL/min; wave length: 0~1.5 min: 400 nm, 1.5~3.5 min: 210 nm; 3.5~4.5 min: 320 nm; 4.5~5.5 min: 203 nm; 5.5~7.0 min: 278 nm; 7.0~9.0 min: 306 nm; 9.0~11.0 min: 285 nm; 11.0~16.0 min: 280 nm. MS conditions: ESI positive ion mode, capillary voltage: 3.3 KV; negative ion mode, capillary voltage: -2.7 KV; ion source temperature: 110 C; dissolvent gas temperature: 350 C; dissolvent gas flow rate: 600 L/hr; conical pore gas flow: 50 L/hr; ion detection mode: full scanning of positive ion and negative ion; ion pair scanning range: 50~1000 m/z.

### 2.3. Animals and Experimental Design

All the procedures conformed to the Guide for the Care and Use of Laboratory Animals published by the National Institutes of Health. Goto-Kakizaki (GK) rats (200 to 300g), purchased from Shanghai Slack Animal Center (certificate no. SCXK (Shanghai) 2012-0002), were used in this experiment. They were raised in specific pathogen-free (SPF) room in North China University of Science and Technology. One-week acclimation later, rats were fed with high glucose and fat for 4 weeks to induce the model of T2DM. Then rats with FBG≥11.1 mmol/L and blood glucose ≥16.7 mmol/L were randomly divided into five groups: the model group (n = 6, oral administration of equivalent volume of normal saline), the metformin group (n = 6, oral administration of metformin, 85 mg/kg/day), WSZYF low-dose group (WSZYF (L), n = 6, oral administration of WSZYF, 300 mg/kg/day), WSZYF medium-dose group (WSZYF (M), n = 6, oral administration of WSZYF, 600 mg/kg/day), and WSZYF high-dose group (WSZYF (H), n = 6, oral administration of WSZYF, 1200 mg/kg/day). Wistar rats orally administered with equivalent volume of normal saline were served as the control group. All the rats were administered for 8 weeks. At the end of the study, indicators related to blood glucose and insulin resistance were measured. Pancreatic tissues were stored at -80°C for further analysis.

### 2.4. Serum Chemistry Assay

Eight weeks later, serum samples were collected for serum chemistry tests. FBG was measured by using blood sugar meter and blood sugar test paper. TG, TC, FFA, and insulin were detected by kits analysis under the manufacture's instruction. Insulin resistance was assessed by a homeostasis model assessment of IR index as previously described [13].

### 2.5. Histological Staining

The normal and changed morphologies of formalin-fixed paraffin-embedded pancreatic tissue sections (4 *μ*m) were detected by hematoxylin and eosin staining.

### 2.6. Cell Culture and Establishment of Apoptosis Model of MIN6 Cells

MIN6 cells line was obtained from Cell Bank of Fudan University (Shanghai, China). The MIN6 cells were cultured in DMEM supplemented with 10% FBS, 100 U/ml penicillin, 100*μ*g/ml streptomycin, and o.4 *μ*l/L*β*-thio ethanol in a humidified incubator with 5% CO2 at 37°C. Cells were divided into several groups: the control group, the model group, the metformin group, and WSZYF groups (H, M, L). When MIN6 cells were cultured to 70-80% confluence, in model group high glucose (25mmol/L) and palmitic acid (0.5 mmol/L) were added and cocultured for 12 hours to induce apoptosis. Cells in metformin and WSZYF groups were treated in the same way, except that 12 hours later, metformin (100 *μ*g/ml) and WSZYF (50, 100, 200*μ*g/ml) were added to the culture with phenol red-free DMEM. In control group, fresh nutrient solution was changed at the time point of 12 hours. The number of biological and technical replicates is 6 in all the vitro experiments.

### 2.7. Cell Viability Analysis

MIN6 cells were seeded in 96-well plates at a density of 5×10^3^ cells per well. When cells were cultured to 70-80% confluence, the model of apoptosis was induced and, 12 hours later, metformin (100 *μ*g/ml) and WSZYF (50, 100, 200*μ*g/ml) were added to the culture for 24 hours. After that, cells viability was determined by MTT assay. Cells were cultured with MTT solution dissolved in DMEM (0.5 mg/ml). Four hours later, the supernatants were removed and 150*μ*l DMSO was added to each well to dissolve the precipitate. Then the absorbance of each well was measured with a microplate reader (TECAN M200PRO, Switzerland) at a wavelength of 492 nm.

### 2.8. Glucose-Stimulated Insulin Secretion

The MIN6 cells were cultured in 24-well plates at a density of 5×10^4^ cells / ml. After different treatments, the supernatants were removed and high glucose (16.7mmol/L) was added to each well for 2 hours to stimulate insulin secretion. Then the supernatants were collected and insulin was measured by insulin assay kit.

### 2.9. Western Blot Analysis on Proteins Related to Apoptosis

Apoptosis-related proteins were detected both by* in vivo* and* in vitro* studies. The collected pancreatic tissues and MIN6 cells were prepared with RIPA buffer (PPLYGEN, China) and proteins were extracted according to the manufacture's instruction. Protein contents were measured with BCA protein assay kit (PPLYGEN, China). After the addition of loading buffer and boiling for 5 minutes, the samples were separated by 10% SDS-PAGE and transferred to NC membranes (Millipore, Germany). Being blocked with 5% nonfat dry milk for 2 hours, the membranes were incubated with different primary antibodies overnight at 4°C. After washing with TBST three times, the membranes were incubated with HRP-conjugated secondary antibodies for 1 hour at room temperature. Then being washed three times with TBST, the proteins were detected with an enhanced chemiluminescence agent (GE, USA) and quantified by densitometry using an image analyzer (Bio-Rad, USA). Mouse anti-GAPDH monoclonal antibody served as an internal control.

The primary antibodies included rabbit monoclonal antibodies against ERK1/2 (1:1000), MEK1/2 (1:1000), p-MEK1/2 (1:1000), and p-ERK1/2 (1:1000); rabbit polyclonal antibody against cleaved-caspase-3 (1:1000); mouse monoclonal antibody against GAPDH (1:5000); and secondary antibodies (goat anti-rabbit, 1:5000; goat anti-mouse, 1:5000).

### 2.10. Statistical Analysis

Data were presented as mean ± standard deviation (SD). Statistical analysis was undertaken by one way analysis of variance (ANOVA) and least significant difference (LSD) post hoc analysis. Differences between groups were considered as statistically significant when* P*< 0.05.

## 3. Results

### 3.1. HPLC Analysis of the Extract

**Figure 1:**
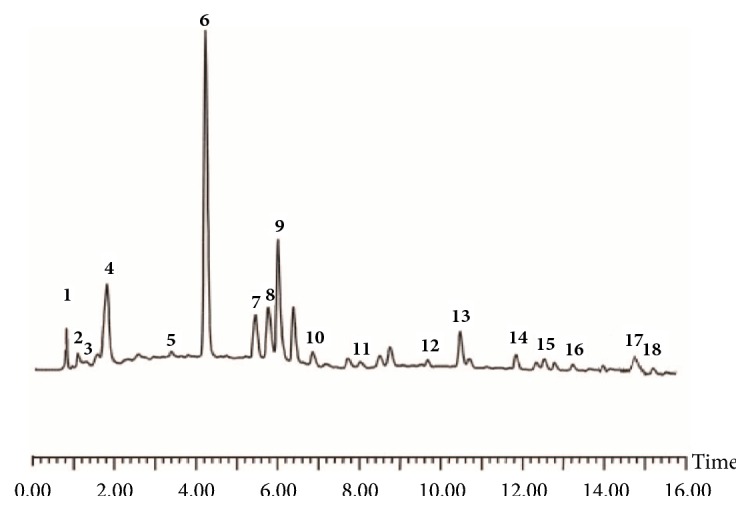
Qualitative analysis on freeze-dried powder of QSKL. HPLC chromatograms numbered from 1 to 18 represent the following: 1 malic acid (PubChem CID:525); 2 citric acid (PubChem CID: 311 ); 3 gallic acid (PubChem CID:370); 4 oleanolic acid (PubChem CID:10494); 5 ursolic acid (PubChem CID:64945); 6 2,3,5,4′-tetrahydroxystilbene-2-o-beta-d-glucopyranoside (PubChem CID:5321884); 7 l-deoxynojirimycin (PubChem CID:29435); 8 cassiaside (PubChem CID:164146); 9 rubrofusarin gentiobioside (PubChem CID:503733); 10 cassiaside B (PubChem CID:131752379); 11 resveratrol (PubChem CID:445154); 12 physcion 8-glucoside (PubChem CID:5319323); 13 aurantio-obtusin (PubChem CID:155011); 14 chrysoobtusin (PubChem CID:155381); 15 obtusifolin (PubChem CID:3083575); 16 emodin (PubChem CID:3220); 17 chrysophanol (PubChem CID:10208); 18 icaritin (PubChem CID:5318980).

HPLC fingerprint chromatograms of WSZYF were shown in Figure 1. Eighteen compounds in the extract were well identified. The compounds were as follows: malic acid (PubChem CID:525), citric acid (PubChem CID:311), gallic acid (PubChem CID:370), oleanolic acid (PubChem CID:10494), ursolic acid (PubChem CID: 64945), 2,3,5,4′-tetrahydroxystilbene-2-o-beta-d-glucopyranoside (PubChem CID: 5321884), l-deoxynojirimycin (PubChem CID:29435), cassiaside (PubChem CID:164146), rubrofusarin gentiobioside (PubChem CID:503733), cassiaside B (PubChem CID:131752379), resveratrol (PubChem CID:445154), physcion 8-glucoside (PubChem CID:5319323), aurantio-obtusin (PubChem CID:155011), chrysoobtusin (PubChem CID:155381), obtusifolin (PubChem CID:3083575), emodin(PubChem CID:3220), chrysophanol (PubChem CID:10208), and icaritin (PubChem CID:5318980).

### 3.2. Effects of WSZYF on Fasting Blood Glucose and Insulin Sensitivity

**Figure 2:**
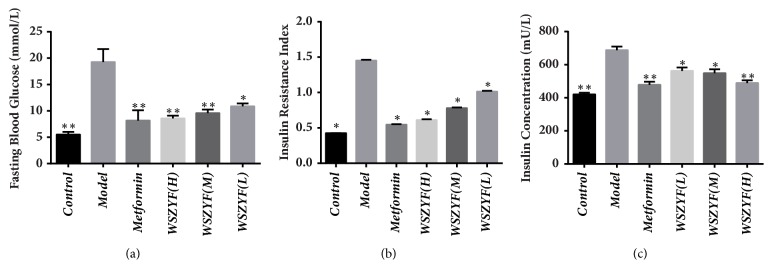
Effects of WSZYF on fasting blood glucose and insulin sensitivity.** (a)** Fasting blood glucose.** (b)** Insulin resistance index.** (c)** Insulin concentration.  ^*∗*^*P* < 0.05,  ^*∗∗*^*P* < 0.01 compared with model group.

*In vivo*, as shown in Figure 2, FBG, insulin concentration, and IR index increased remarkably in T2DM model group. These three indicators demonstrated that glucose metabolism was disrupted and IR occurred. Treatments of WSZYF and metformin lowered all the indexes mentioned above, indicating that they could restore glucose metabolism disorders and improve the insulin sensitivity.

### 3.3. Effects of WSZYF on Lipid Metabolic Parameters

**Figure 3:**
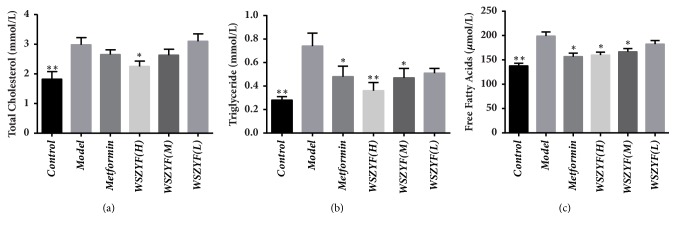
Effects of WSZYF on lipid metabolic parameters.** (a)** Total cholesterol.** (b)** Triglyceride.** (c)** Free fatty acids.  ^*∗*^*P* < 0.05,  ^*∗∗*^*P* < 0.01 compared with model group.

In the model group, TC, TG, and FFA were all significantly elevated (Figure 3), indicating that hyperlipidemia symptoms were also successfully established in the T2DM group. Treatment with metformin and WSZYF in a medium dose (WSZYFM) dramatically reduced TG and FFA levels in serum, but not for TC. WSZYFH (high dose) could decrease the levels of all the three indexes mentioned above, but the low dose of WSZYF had no effect on lipid metabolic parameters.

### 3.4. Effects of WSZYF on the Morphology and Structure of Pancreatic Tissues

**Figure 4:**
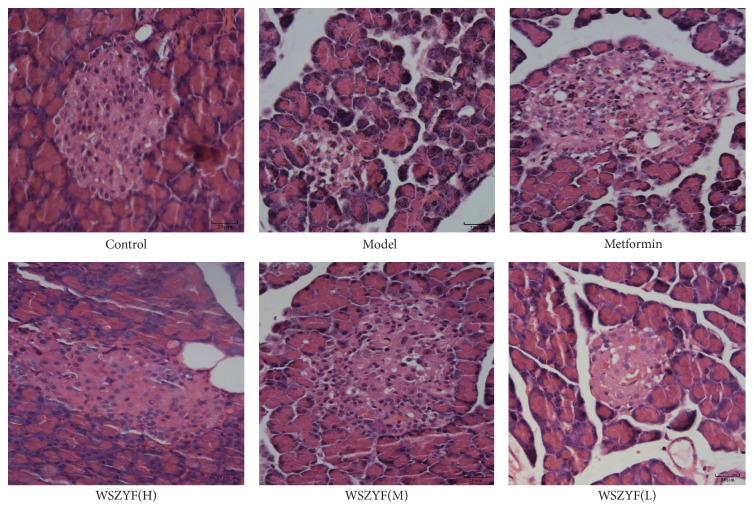
Effects of WSZY on the morphology and structure of islets.

The hematoxylin-eosin (HE) staining was used to observe the changes of pancreatic tissues. Findings under the microscope suggested that there was severe injury to the pancreas in T2DM rats. For example, the number of pancreatic cells decreased, and the diameter of pancreatic islet was shortened. Meanwhile, the structure of the pancreatic islet was not in order with emerging vacuoles and swollen nuclei. Both metformin and low, medium, and high dose of WSZYF could significantly reduce all the indexes mentioned above related to pancreatic injury, and the effect was best in WSZYFH (Figure 4).

### 3.5. Effects of WSZYF on Cell Viability

**Figure 5:**
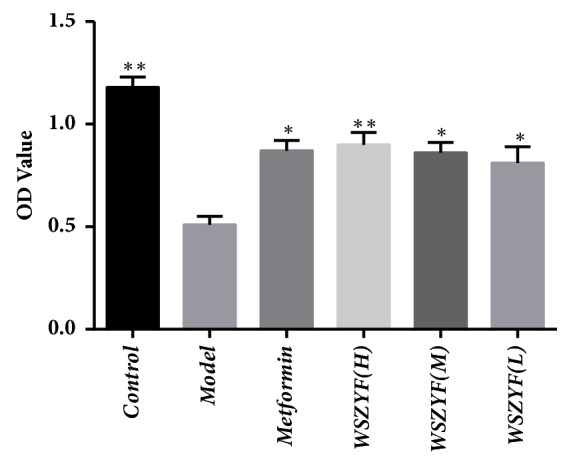
Effects of WSZYF on cell viability.  ^*∗*^*P* < 0.05,  ^*∗∗*^*P* < 0.01 compared with model group.

Cell viability was assessed by MTT assay. The optical density (OD) value was used to represent viability. As shown in Figure 5, OD value in the model group was lower than that in the control group, indicating that high glucose and palmitic acid significantly promoted cytotoxicity in MIN6 cells. The effects of metformin and WSZYF on cell viability were also detected. Compared with the model group, OD values in the treatment groups were higher, demonstrating they provided significant protective effects on cells viability.

### 3.6. WSZYF Enhanced Glucose-Stimulated Insulin Secretion

**Figure 6:**
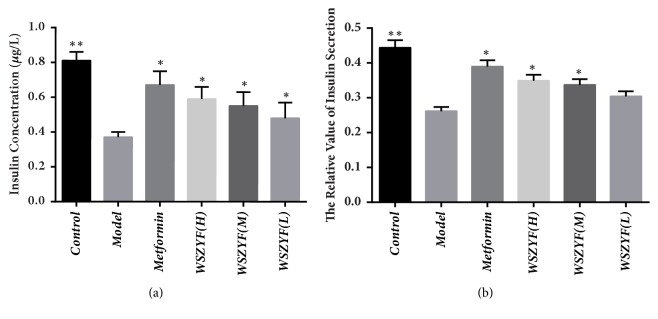
WSZYF enhanced glucose-stimulated insulin secretion.** (a)** Insulin concentration.** (b)** The relative value of insulin secretion.  ^*∗*^*P* < 0.05,  ^*∗∗*^*P* < 0.01 compared with model group.

Insulin secretion stimulated by high glucose was selected as an index to evaluate the effect on promoting insulin secretion of WSZYF in MIN6 cells. The insulin concentration in the model group was lower than that in the control group, while in treatment groups, insulin concentrations were significantly elevated (Figure 6(a)). Considering the different cell activities among groups, relative value of insulin concentration was used to express insulin secretion. In the model group, MIN6 cells were in low response to the stimulation of high glucose. The medium and high dose of WSZYF and metformin treatments promoted insulin secretion of MIN6 cells stimulated by high glucose (Figure 6(b)).

### 3.7. Effects of WSZYF on the Expressions of Apoptosis-Related Proteins

**Figure 7:**
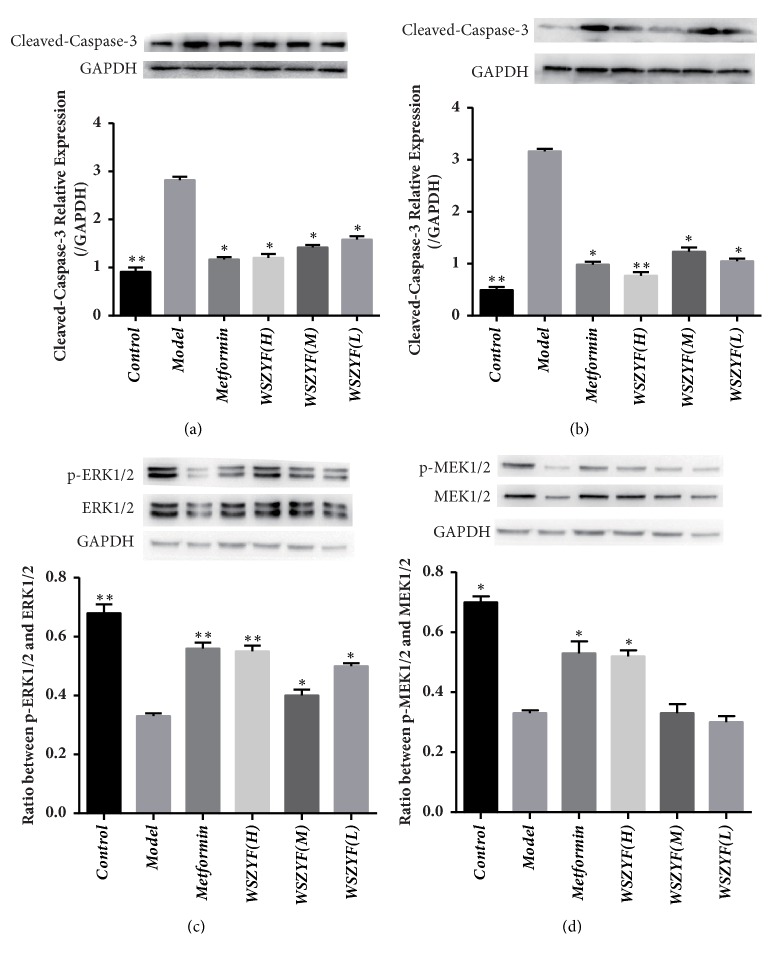
Effects of WSZYF on the expressions of apoptosis-related proteins.** (a)** The relative expression of cleaved-caspase-3* in vivo*.** (b)** The relative expression of cleaved-caspase-3* in vitro*.** (c)** The ratio between p-ERK1/2 and ERK1/2* in vitro*.** (d)** The ratio between p-MEK1/2 and MEK1/2* in vitro*.  ^*∗*^*P* < 0.05,  ^*∗∗*^*P* < 0.01 compared with model group.

In the following, the mechanism of WSZYF in protecting MIN6 cells injury was investigated. Expressions of the molecules related to apoptosis were measured by Western blot. As shown in Figure 7, in the model group, the ratios of p-ERK1/2 and ERK1/2, p-MEK1/2 and MEK1/2 decreased, whereas the expression of cleaved-caspase-3 (both* in vivo* and* in vitro*) increased compared to the control group, indicating that apoptosis was activated. Treatment with metformin and WSZYF reversed the expressions of these proteins back toward normal levels, except that WSZYF (in medium and low dose) had no effect on the ratio between p-MEK1/2 and MEK1/2. The results showed that protection of WSZYF against MIN6 cells apoptosis might be modulated through MEK-ERK-Caspase-3 signaling pathway.

## 4. Discussion

Dysfunction and apoptosis of pancreatic *β* cell have proved to be vital in the occurrence and development of IR in T2DM. In this study, we demonstrated that WSZYF could exert antidiabetic effect by inhibiting pancreatic *β* cell apoptosis* in vivo* and* in vitro*. Further experiment indicated that the antiapoptotic effect of WSZYF potentially occurred via regulating MEK-ERK-Caspase-3 signaling pathway.

Traditional Chinese medicine makes great contributions in the treatment of T2DM.* Wushenziye* formula was widely applied clinically and had proved to improve IR in skeletal muscle [12]. In this study, we made the chemical analysis of WSZYF by HPLC. Eighteen compounds in the extract were well identified. Among them, gallic acid, oleanolic acid, and resveratrol have been reported to play different roles in the treatment of T2DM [14–16]. For example, gallic acid, a major composition of Tibetan Medicine Tang-Kang-Fu-San, improved the abnormal pathological changes in pancreas tissues [14]. All the above provided experimental evidence for WSZYF to protect the pancreas in the treatment of T2DM.

T2DM is a multiple link metabolic disorder syndrome. With in-depth research, it is clear that the dysfunction of pancreatic *β* cell is one of the key factors in T2DM [2]. The function of pancreatic *β* cell is progressively impaired in the progression of T2DM. The autopsy reports also confirmed that the number of pancreatic *β* cell in T2DM patients was significantly reduced [17]. It has been reported that apoptosis is a key factor in leading to dysfunction of pancreatic *β* cell, which triggers insufficient insulin secretion [18, 19]. Therefore, increase of number and functional protection in pancreatic *β* cell have become one of the key measures to improve T2DM. In our study, FBG, insulin concentration, IR index, TG, TC, and FFA increased dramatically in the model group, indicating that glucolipotoxicity and insulin resistance occurred in T2DM. Further HE staining showed that islets suffered from severe injury, expressed as decreased pancreatic cells, diminished diameter of pancreatic islets, disordered structure of the pancreatic islets, appearing vacuoles, and swollen nuclei. Compared with the model group, WSZYF could reduce all the levels of the indicators mentioned above and improve the morphology and structure of the pancreatic tissues. These findings verify that WSZYF is effective in protecting pancreatic tissues, attenuating glucolipotoxicity and ameliorating IR.

Glucolipotoxicity plays a pivotal role in the dysfunction of pancreatic islet [20]. In the early stage, the persistence of hyperglycemia and hyperlipidemia will aggravate IR and further weaken the function of insulin secretion, which are displayed as the reduction of insulin secretion and the decrease of pancreatic *β* cells [21, 22]. They affect each other and lead to a vicious circle. Based on these, we chose high glucose and palmitic acid to induce apoptosis model of MIN6 cells. In the model group, the viability of MIN6 cells decreased dramatically and the secretion of insulin stimulated by high glucose decreased. All of these results further demonstrated the toxic effects of high glucose and palmitic acid on MIN6 cells. After WSZYF treatment, cell viability enhanced and cells showed good responses to high glucose stimulation, revealing that WSZYF is effective in inhibiting toxic activity to pancreatic *β* cells caused by high glucose and palmitic acid.

Apoptosis caused by glucolipotoxicity is the main reason for the decreased pancreatic *β* cells in T2DM [23]. MAPK signaling pathway plays an important role in the process of pancreatic *β* cells' proliferation, transformation, differentiation, and apoptosis [24, 25]. The MEK-ERK signaling pathway is one of the classic MAPK signaling transduction pathways and accomplishes the transduction through three cascade reactions of MAPK. It participates in mitosis signals, regulates cell cycle signals, and determines cell differentiation and apoptosis [26, 27].The cascade activated Ras-Raf-MEK-ERK signaling pathway ultimately downregulates caspase-3 expression, which acted as a proapoptotic factor, and inhibits apoptosis [28, 29]. In this study, Western blot was applied to detect apoptosis-related proteins. In the model group, the expressions of cleaved-caspase-3 increased both* in vivo* and* in vitro*. Meanwhile, MEK1/2, ERK1/2, p-MEK, and p-ERK1/2 were captured in lower expressions. Treatment with WSZYF make the proteins return to normal expressions, except that the medium and low dose of WSZYF had no effect on the ratio between p-ERK1/2 and ERK1/2. It is indicated that WSZYF can inhibit apoptosis of the pancreatic *β* cells caused by glucolipotoxicity through the modulation of the MEK-ERK-Caspase-3 signaling pathway.

## 5. Conclusions

In summary, we investigated the effects of WSZYF in T2DM animal model and apoptosis model of MIN6 cells in this study. The results suggested that WSZYF could improve IR by inhibiting pancreatic *β* cell apoptosis through regulating MEK-ERK-Caspase-3 signaling pathway. This study laid a foundation for the clinical application and development of therapeutics.

## Data Availability

The data used to support the findings of this study are available from the corresponding author upon request.
